# Occupational risk of tuberculosis transmission in a low incidence area

**DOI:** 10.1186/1465-9921-6-35

**Published:** 2005-04-14

**Authors:** Roland Diel, Andreas Seidler, Albert Nienhaus, Sabine Rüsch-Gerdes, Stefan Niemann

**Affiliations:** 1 School of Public Health, University of Düsseldorf, Germany; 2 Institute of Occupational Medicine, University of Frankfurt, Germany; 3 Institution for statutory accident insurance and prevention in the health and welfare services, Hamburg, Germany; 4 National Reference Center for Mycobacteria, Forschungszentrum Borstel, Germany

**Keywords:** tuberculosis epidemiology

## Abstract

**Background:**

To investigate the occupational risk of tuberculosis (TB) infection in a low-incidence setting, data from a prospective study of patients with culture-confirmed TB conducted in Hamburg, Germany, from 1997 to 2002 were evaluated.

**Methods:**

*M. tuberculosis *isolates were genotyped by IS*6110 *RFLP analysis. Results of contact tracing and additional patient interviews were used for further epidemiological analyses.

**Results:**

Out of 848 cases included in the cluster analysis, 286 (33.7%) were classified into 76 clusters comprising 2 to 39 patients. In total, two patients in the non-cluster and eight patients in the cluster group were health-care workers. Logistic regression analysis confirmed work in the health-care sector as the strongest predictor for clustering (OR 17.9). However, only two of the eight transmission links among the eight clusters involving health-care workers had been detected previously. Overall, conventional contact tracing performed before genotyping had identified only 26 (25.2%) of the 103 contact persons with the disease among the clustered cases whose transmission links were epidemiologically verified.

**Conclusion:**

Recent transmission was found to be strongly associated with health-care work in a setting with low incidence of TB. Conventional contact tracing alone was shown to be insufficient to discover recent transmission chains. The data presented also indicate the need for establishing improved TB control strategies in health-care settings.

## Background

In recent years, several population-based studies – e.g. in Europe or the USA [1-5] – have, by applying both classical epidemiological and molecular strain-typing techniques, revealed a high frequency of transmission of tuberculosis (TB), even in countries with a low TB incidence. *Mycobacterium tuberculosis *strains with DNA fingerprint patterns that are identical in respect of the insertion sequence IS*6110 *indicate possible transmission chains, and on the basis of this it has been concluded [6] that recently transmitted infections with rapid progress to active TB seem generally to play an important part in the spread of TB. However, there is a lack of information on the contribution of recent transmission to occupationally acquired TB. Up to now, only a few studies have been performed that have applied modern molecular DNA-fingerprint techniques capable of tracing directly routes of transmission attributable to occupational exposure, e.g. among health-care workers [5,7-9].

For Germany, no prospectively acquired data on this topic have been available up to now. The incidence of TB in Germany is relatively low and steadily decreasing, with a total of 7866 new cases reported to the Robert Koch Institute in 2001 (9.6 per 100,000 inhabitants [10]). However, in the city of Hamburg (with its 1.7 million residents the second largest city in Germany) the overall incidence was 16.3 cases per 100,000 in 2001 and, against the national downward trend, is currently rising [11]. In 2001, as in previous years, Hamburg had a TB incidence rate higher than in any of the other fifteen German federal states.

In order to identify the pathways of TB transmission and to determine the predictors of clustering of identical isolates in this metropolis, a long-term, prospective, population-based molecular-epidemiological study has been in progress in Hamburg since 1 January 1997 and is ongoing. As a preliminary result of the first three study years (1997–1999) referring to a sample of only 398 culturally proven TB patients it could be shown that conventional contact tracing prior to IS*6110 *RFLP analysis by far underestimates the amount of recent TB transmission in a metropolis like Hamburg. In Germany – as well as in most other European countries – profession is not declared when TB disease is reported; thus the excess risk for health-care workers over members of the general population is unknown. Because precise data on the occupational risk of TB infection in low incidence settings are urgently needed for the development of better-aimed TB control, this study – now comprising more than twice as many patients within an observation period of six years – should help to evaluate the risk of recently transmitted disease in health-care settings.

## Methods

### Study population

The study includes all patients in Hamburg in whom *M. tuberculosis *complex is confirmed by culture ("definite cases" [12]) as reported to each of the seven district public health departments from 1 January 1997 with a cut-off for the present analysis of 31 December 2002. Case data were collected prospectively by trained public-health staff using a standardised questionnaire. By interviewing each patient, information was obtained on: the patient's sex, country of birth, nationality, immigration status (if applicable), current address (or whether the patient was homeless), the nature of the patient's current employment (if any), details of any previous known exposure to other persons with tuberculosis. The subgroup of health-care workers refers to all the paid and unpaid persons working in health-care settings and potentially exposed to *M. tuberculosis *[13].

To acquire clinical data, the following were also included in the questionnaire: date of first onset of illness, date and reason for the diagnostic investigation, latency due to the patient's delay in seeking medical help, associated medical problems (especially HIV infection), chest radiographic findings, results of microbiological analyses and presence of alcoholism (according to the WHO-ICD 10-classification [14]).

### IS*6110*-RFLP analysis

Extraction of DNA from mycobacterial strains and DNA fingerprinting using IS*6110 *as a probe were performed according to standard protocols [15,16]. The IS*6110 *fingerprint patterns of mycobacterial strains were analysed by using the Bionumerics software (Applied Maths, Kortrijk, Belgium) as described previously [17-19]. Clusters were defined as groups of patients with *M. tuberculosis *strains showing identical RFLP patterns. Patients with isolates with fewer than five bands were not included in this study.

### Statistical analysis

All patients included were classified into two groups, "clustered" and "non-clustered". Categorical data were compared by the chi-square test (or Fisher's exact test). Wilcoxon's rank sum test was performed to determine whether the distribution of treatment duration as a continuous variable differed between two groups. All tests were performed as two-sided tests. *P *values below 0.05 were considered significant.

### Potential confounders and statistics

Odds ratios (OR) and 95% confidence intervals (CI) were calculated by using logistic regression analysis. As tuberculosis patients aged less than 16 years (n = 22) could not contribute to the analysis of occupationally transmitted tuberculosis, they were excluded from the analysis of the relationship between specific outcome variables and clustering. The following potential confounders were judged separately with respect to their influence on the OR for health-care work: age; sex; AIDS; foreign birth; unemployment; homelessness; alcohol abuse; drug addiction; previous history of tuberculosis; sputum smear positivity; latency period between first symptoms and TB diagnosis; history of contact tracing. Variables were included in the final regression model for health-care work if they changed the odds ratio for health-care work by 10% or more. Therefore, we kept the following confounders in the final regression model for health-care work: foreign birth; unemployment; alcohol abuse; drug addiction; the latency period between first symptoms and TB diagnosis. The OR for variables other than health-care work were adjusted for the same variables that had been included in the final regression model for health-care work. For variables other than health-care work, the selection of confounders started with the final regression model for health-care work. Afterwards, for each variable other than health-care work, the following potential confounders were judged separately with respect to their influence on the odds ratio: age; sex; AIDS; homelessness; previous history of tuberculosis; sputum smear positivity; history of contact tracing. Non-health-care variables were included in the regression model if they changed the odds ratio for the specific outcome variable by 10% or more. According to this selection strategy, the ORs for homelessness and previous history of TB were additionally adjusted for history of contact tracing; the OR for drug addiction was additionally adjusted for AIDS.

## Results

Up to 31 December 2002, 895 cases of pulmonary and 66 cases of non-pulmonary tuberculosis reported to the public health offices were identified as culture-positive for *M. tuberculosis *complex. Culture isolates from 848 patients' isolates were available for RFLP fingerprinting (88.2%). Their relevant characteristics are given in Table 1.

**Table 1:** Univariate analysis of risk factors for patients belonging to IS*6110 *RFLP clusters

**Characteristic**	**Non-cluster group**	**Cluster group**	**All patients**	***p***
	**(N = 562)**	**(N = 286)**	**(N = 848)**	
Age (yr) mean ± SD	44.9 ± 19.7	43.2 ± 17.2	44.4 ± 18.9	n.s.
AIDS (n, %)	27 (4.8)	21 (7.3)	48(5.7)	n.s.
Resistance to any drug (n, %)	52 (9.3)	11 (3.8)	63 (7.4)	0.005
MDR (n, %)	6 (1.1)	4 (1.4)	10 (1.2)	n.s.
Foreign-born (n, %)	270 (48.0)	96 (33.6)	366 (43.2)	<0.001
Male (n, %)	335 (59.6)	190 (66.4)	525 (61.9)	n.s.
Female (n, %)	227(40.4)	96 (33.6)	323 (38.1)	n.s.
Drug abuse (n, %)	30 (5.3)	40 (14.0)	70 (8.3)	<0.001
Alcohol abuse (n, %)	80 (14.2)	113 (39.5)	193 (22.8)	<0.001
Homelessness (n, %)	41 (7.3)	29 (10.1)	70 (8.3)	n.s.
History of contact tracing (n, %)	42 (7.5)	70 (24.5)	112 (13.2)	<0.001
Site of tuberculosis				
- Pulmonary (n, %)	513 (91.3)	268 (93.7)	781 (92.1)	n.s.
- Extrapulmonary (n, %)	49 (8.7)	18 (6.3)	67 (7.9)	n.s.
- Cavitary disease (n, %)	109 (19.4)	93 (32.5)	202 (23.8)	<0.001
Sputum smear positivity (n, %)	170 (30.2)	118 (41.3)	288 (34.0)	0.001
Previous history of Tb (n, %)	62 (11.0)	43 (15.0)	105 (12.4)	n.s.
Diagnosis due to symptoms (n, %)	492 (87.5)	186 (65.0)	678 (80.0)	<0.001
Unemployment (n, %)	108 (19.2)	163 (57.0)	271 (32.0)	<0.001
Contact persons with disease (n, %)	5 (8.9)	60 (21.0)	65 (7.7)	<0.001
Diagnosis due to contact tracing (n, %)	8 (1.4)	30 (10.5)	38 (4.5)	<0.001
Health-care worker (n, %)	2 (0.4)	8 (2.8)	10 (1.2)	<0.004
Latency from first symptoms to diagnosis				
< 6 months (n, %)	444 (79.0)	164 (57.3)	608 (71.7)	<0.001
>= 6 months; < 12 months (n, %)	98 (17.4)	79 (27.6)	177 (20.9)	<0.001
>= 12 months (n, %)	20 (3.6)	43 (15.0)	63 (7.4)	<0.001

n.s., not significant

The average age was 44.4 ± 18.9 years (mean ± SD; Table 1). The majority of patients were male (525/848; 61.9%). In the study population, 366 patients (43.2%) were born outside Germany; 48/848 (5.7%) showed clinical symptoms of HIV infection (AIDS); 70 (8.3%) were homeless at the start of the study; 193 (22.8%) appeared to be alcohol-dependent; 70 (8.3%) regularly injected drugs of abuse; 105 (12.4%) had a past history of TB; 112 (13.2%) had been previously identified as contact persons of infectious TB sources; 63 (7.4%) showed resistance to antituberculotic drugs, but of these only 10 (1.2%) cases were reported with resistance to at least INH and RIF, and were thus multidrug-resistant.

Two hundred and eighty-six patients (33.7%) shared an identical RFLP pattern with one or more other patients and were thus classified, as clustered cases, into 76 clusters ranging in size from 2 to 39 persons. Among the clusters, 39 (51.3%) comprised only two patients each, thus representing 78/146 (53.4%) of the cluster patients.

### Characterisation of cluster patients and identification of risk factors associated with clustering

In order to identify significant differences between the 286 patients in clusters and the 562 patients not in clusters, univariate analyses were performed. The results for the different variables analysed are summarised in Table 1.

There was no significant difference in age distribution between the two groups, as determined by Wilcoxon rank sum test (*p *= 0.63). Chi-square tests showed that the patients in clusters were more likely than the non-clustered patients to be drug abusers (*p *< 0.001), to be alcoholics (*p *< 0.001), to be health-care workers (*p *= 0.004), to be unemployed (*p *< 0.001), to be sputum-smear positive (*p *= 0.001) and to have a cavitary disease (*p *< 0.001), and they had more known contacts to patients with active tuberculosis (*p *< 0.001). A history of involvement in the contact tracing of tuberculosis patients was also significantly associated with clustering (*p *< 0.001). In the "cluster" group fewer patients were diagnosed because of symptomatic disease (*p *< 0.001), but more were found through contact tracing, irrespective of symptoms (*p *< 0.001). Drug resistance was more common in patients not found in a cluster (*p *= 0.005), and the proportion of foreign-born patients was smaller among the clustered patients (*p *< 0.001). The latency period between the first onset of symptoms and confirmed diagnosis was far longer in cluster patients (*p *< 0.001).

In multivariate analyses (Table 2), the OR for employment as a health-care worker was found to be the strongest predictor (OR = 17.9; CI 3.6–89.3), followed by a latency period between the first onset of symptoms and confirmed diagnosis of at least 12 months (OR = 7.5: CI 3.7–13.9) or of at least 6 months (OR = 6.0; CI 3.4–10.4), unemployment (OR = 5.2; CI 3.6–7.5) and a history of contact tracing (OR = 2.8; CI 1.7–4.6). Alcoholism – the leading predictor in our first evaluation three years ago [6] – represented a further considerable risk of being in a cluster (OR = 2.6; CI 1.7–3.8), whereas drug addiction (OR = 1.7; CI 1.0–3.1), sputum smear positivity (OR = 1.2; CI 0.9–1.8), AIDS (OR = 0.8; 0.4–1.8), male sex (OR = 1.1; CI 0.6–1.8), foreign origin (OR = 0.5; CI 0.3–0.7) and age (see the results of several groups; Table 2) were not significant independent risk factors.

**Table 2:** Health-care work and clustering (multivariate analysis).

**Variable**	**Non-clustered TB**		**Clustered TB**		**Crude OR**	**95% CI**	**Adj. OR^b^**	**95% CI**
	**N^a^**	**%**	**N^a^**	**%**				
Health-care worker								
No	545	99.6	269	97.1	1.0	-	1.0	-
Yes	2	0.4	8	2.9	8.1	1.7–38.4	17.9	3.6–89.3
AIDS								
No	520	95.1	256	92.4	1.0	-	1.0	-
Yes	27	4.9	21	7.6	1.6	0.9–2.8	0.8	0.4–1.8
Foreign-born								
No	284	51.9	185	66.8	1.0	-	1.0	-
Yes	263	48.1	92	33.2	0.5	0.4–0.7	0.5	0.3–0.7
Sex								
Female	221	40.4	90	32.5	1.0	-	1.0	-
Male	326	59.6	187	67.5	1.4	1.0–1.9	1.1	0.8–1.6
Drug abuse								
No	517	94.5	237	85.6	1.0	-	1.0	-
Yes	30	5.5	40	14.4	2.9	1.8–4.8	1.7	1.0–3.1
Alcohol abuse								
No	468	85.6	164	59.2	1.0	-	1.0	-
Yes	79	14.4	113	40.8	4.1	2.9–5.7	2.6	1.7–3.8
Homelessness								
No	510	93.2	249	89.9	1.0	-	1.0	-
Yes	37	6.8	28	10.1	1.6	0.9–2.6	0.9	0.5–1.7
History of contact tracing								
No	505	92.3	211	76.2	1.0	-	1.0	-
Yes	42	7.7	66	23.8	3.8	2.5–5.7	2.8	1.7–4.6
Sputum smear positivity								
No	377	68.9	161	58.1	1.0	-	1.0	-
Yes	170	31.1	116	41.9	1.6	1.2–2.2	1.2	0.9–1.8
Previous history of TB								
No	485	88.7	234	84.5	1.0	-	1.0	-
Yes	62	11.3	43	15.5	1.4	0.9–2.2	1.7	1.0–2.7
Unemployment								
No	441	80.6	114	41.2	1.0	-	1.0	-
Yes	106	19.4	163	58.8	5.9	4.3–8.2	5.2	3.6–7.5
Latency period from first symptoms to TB diagnosis								
< 6 months	432	79.0	156	56.3	1.0	-	1.0	-
≥ 6 months; <12 months	95	17.4	78	28.2	2.3	1.6–3.2	6.0	3.4–10.4
≥ 12 months	20	3.7	43	15.5	2.6	1.7–3.8	7.5	3.7–13.9

^a ^TB patients < 16 years (n = 22) excluded from the analysis

^b ^Adjusted for foreign birth; unemployment; alcohol abuse; drug addiction; and the latency period between first symptoms and TB diagnosis; the odds ratios for homelessness and previous history of TB are additionally adjusted for history of contact tracing; the odds ratio for drug addiction is additionally adjusted for AIDS

### Epidemiological analysis of clustered cases and efficiency of classical contact tracing

**Figure 1:**
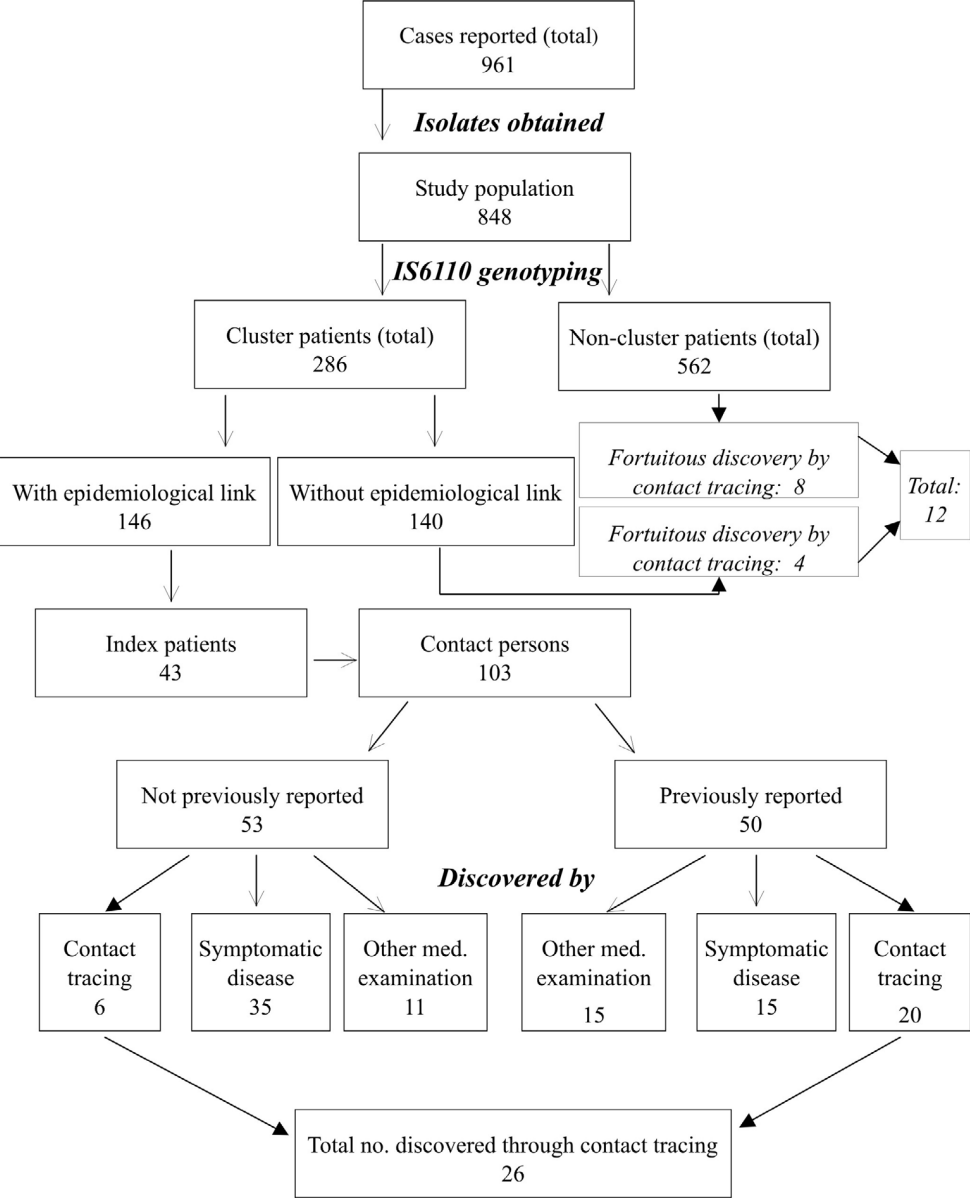
Distribution of cases discovered by contact tracing and alternative sources

As in our preliminary study [6], in addition to acquiring data from standardised patient questionnaires, we also performed intensive epidemiological investigations, including additional interviews for patients within clusters. Recent transmission, verified by epidemiological relationships, could be confirmed for 146 of the 286 clustered patients (51.0%) in 35 of the 76 clusters (46.1%). Of these 146 patients, 43 were source cases suffering from tuberculosis, labelled as index persons for the purpose of the investigation and identified by earlier onset of disease, and 103 were infected by these persons, with onset of disease during the study period (Figure 1); 50 of the 103 (49%) had already been reported as contact persons to the public health bureaus. Only 20 of these 50 (40%) had been identified by contact tracing procedures; a further 15 (30%) had independently sought medical attention because of symptoms, and the remaining 15 (30%) were identified by other means (screening as part of the asylum procedures, or diagnostic measures performed because of other diseases).

Fifty-three patients could only be identified retrospectively, by RFLP fingerprinting, as contact persons of their respective index cases. Of these, six were discovered to be tuberculosis patients through contact tracing (owing to index persons other than their respective index cases), 36 by their symptomatic disease and 11 as a consequence of other medical examinations. In total, conventional investigation of the patients' contacts conducted before RFLP typing identified only 26 (25.2%) of the 103 clustered patients who had became ill between exposure and contact tracing as contact persons with confirmed epidemiological links.

Conversely, contact tracing led to the discovery among the cluster patients of four TB patients with no known epidemiological connection to the other members of their cluster, and also to the fortuitous discovery of eight infected persons who had been named as contact persons but were not members of any cluster. This means, remarkably, that contact tracing based on retrospectively incorrect information in terms of molecular-epidemiological links led to the detection of almost one-half (12/26) as many cases of disease as the contact tracing that was (correctly) rooted in an epidemiological context.

Eight (5.5%) of the 146 cluster patients with an epidemiological connection to others in the study population were employees in the health-case sector, as will be described below. Apart from these cases there were two other patients with sporadic TB, i.e. due to reactivation rather than to recent transmission, not cluster members; both were nurses (a 28-year-old Rumanian and a 52-year-old Russian) who might have been infected as children in their country of origin.

### Description of the cluster relationships

Out of 10 health-care workers in this study 8 were in the cluster group. A short description of these cases is given below:

#### Cluster A

In August 1996, a 32-year-old homeless alcoholic in the intensive-care ward of hospital 1 was diagnosed as having sputum-positive, progressive tubercular pneumonia. He died of multiple causes seven weeks after admission. He had named four close contact persons from the time before his admission; none of these developed disease. Eight months later, in March 1997, a 27-year-old nurse working in the same intensive-care ward was diagnosed as having tubercular pleuritis. In a routine examination of staff, which took place shortly before the admission of the patient who later died, the result of a Mendel-Mantoux intracutaneous test (10TU) had been negative. The cluster analysis revealed the causal relationship: the cluster contained only these two patients. No connection between the deceased patient and the infection of the nurse – a classic instance of fresh transmission – had been made, either by the physician responsible for the staff or by the public health authority in the search for the source of infection. Only afterwards was the occupational health investigation required by German law (BK3101) carried out.

#### Cluster B

On 9 September 1997, a 34-year-old unemployed alcoholic, admitted to hospital 2 for investigation of pneumonia of unknown origin, was found to have sputum-positive pulmonary tuberculosis. He had already been examined in three separate contact tracings between 14 August 1995 and 21 February 1996, as a close contact person of another unemployed alcoholic with known TB, but the result had each time been negative. He was nursed between 9 September and 16 September 1997 by a 33-year-old male nurse, who seven months later became ill with tubercular pleuritis. The required occupational health investigation was initiated, and the alcoholic patient could be assumed to be the most likely source of disease before the result of the RFLP analysis became known. However, it was found that the cluster was not restricted to these two cases; on the contrary, by September 2002 its known membership had increased to nine. Of the remaining seven members, all with pulmonary TB, six were alcoholics and frequented various bars, although no epidemiological connection between these could be established. The seventh was a Rumanian prostitute had lived in Hamburg illegally for only a few months and who had presumably been infected by a client.

#### Cluster C

A 67-year-old ear, nose and throat (ENT) specialist became ill in May 2002 with culturally confirmed pulmonary TB. In his view no connection with any former patient could explain this. RFLP analysis revealed a strain identical to that in a Nepali waiter who three years earlier, in May 1999 – then 21 years of age – had received out-patient treatment for earache from the same doctor. As he had only visited this doctor before the TB diagnosis and had not mentioned him as a contact person, the connection could only be established retrospectively.

#### Cluster D

An 86-year-old woman was admitted to hospital 3 with fever of unknown cause. During the routine diagnostic procedures, in early September 2002, a medical technician in the hospital laboratory pricked herself in the right index finger with a sharp metal object after withdrawing incubated material from a liquid-culture bottle. The infection was followed up in an occupational health investigation and, because of the clear causality, was registered as being work-related infection before the RFLP analysis was performed. On 12 September the patient was diagnosed as having urogenital TB. Shortly after this the technician developed a protracted skin granuloma that was found to be tubercular on 6 November.

#### Cluster E

In April 2000 a 73-year-old patient in the ENT department of hospital 4 received a tympanoplasty because of chronic otitis media. A smear test for TB bacteria was not performed at the time, and an infiltrate in the upper left pulmonary lobe, detected by X-ray, was not investigated. In early July 2000 the patient was admitted to the abdominal surgery ward of the same hospital with an abdominal aortic aneurism. During the pre-operative tests an enlargement of the infiltrate was detected, and two days later sputum-positive pulmonary TB was diagnosed. An ear smear was performed and also found to be TB-positive. Contact tracing was carried out retrospectively on the medical and nursing personnel who had regular contact with the patient. On 3 January 2001 a 21-year-old assistant nurse, who had given a negative tuberculin skin test in the autumn of 1999, now gave a positive tuberculin test and also tested culturally positive for pulmonary tuberculosis.

#### Cluster F

In late February 1998 a 26-year old homeless man with an i.v. drug addiction was admitted to hospital 5 with interstitial pneumonia and general respiratory failure. He was given mechanical respiration in the intensive-care unit. In November 1996 he was diagnosed as having an HIV infection (Category B according to the CDC classification). On 4 March 1998 sputum-positive pulmonary TB was diagnosed. In November 2000 the 55-year-old domestic responsible for disinfection of equipment in this ward began to experience increasing weight loss and general weakness. On 7 March 2001, sputum-positive pulmonary TB was found as well. An occupational cause was not suspected at first; only the retrospective investigation on the basis of an RFLP analysis showed that the domestic had shaken out the tubes of the respiration apparatus, which explained the fresh airborne infection.

#### Cluster G

On 30 March 2001 a 43-year-old male geriatric nurse fell ill with culturally confirmed pulmonary TB; initially, no occupational cause was suspected. He belonged to a cluster that comprised five members with no established epidemiological interrelationships; of the other four, three were Africans and one was the German-born daughter of a Nigerian, 30 months old and with pulmonary TB that had been culture-confirmed by a gastric-fluid sample on 15 July 2000. The source of infection of the girl was her father, who was suffering from AIDS-induced encephalopathy; his consequent mental confusion prevented a diagnosis of sputum-positive TB until long after diagnosis on 9 June 2000. The culture from the father could not be fingerprinted, so this patient was at first not considered in the cluster analysis, and only the correspondence between the strain of the daughter with that of the geriatric nurse prompted a revision of the medical history of the father. It was found that the nurse had, during practical training in the ward for general internal diseases at the hospital 6, looked after the father when the latter had been admitted with pneumonia of unknown origin. As the training period had ended before the father's TB was diagnosed, the geriatric nurse had not been included in the contact tracing.

#### Cluster H

In early January 1997, a 58-year-old homeless alcoholic was diagnosed as having sputum-positive pulmonary TB. Treatment was initiated but was repeatedly interrupted; the patient continued to drink and intermittently gave positive sputum cultures. By the end of 2002, eight additional cluster members had been identified, of whom two were known as direct contacts from the initial tracing, while the other seven belonged to the bar milieu of Hamburg's red-light district. On 3 September 1998, a hospital physician at hospital 7 fell ill with tubercular pleuritis. Contact between the index patient and the physician could only be established retrospectively: the two persons had been in contact for a brief period in March 1998 in the admissions ward, where the patient had been admitted for alcohol withdrawal.

## Discussion

The purpose of conducting this population-based epidemiological study was to determine which factors might influence TB transmission in the city of Hamburg, and how these contribute to the community epidemiology of tuberculosis. Available information about the risk of transmission to employees in the medical sector in other cities is at present sparse, and partly contradictory. Therefore, we especially evaluated the occupational risk of TB infection performed an in depth investigation of the transmissions identified.

In the classical study by Small *et al*. [5], previous care in a tuberculosis clinic was included as a risk variable and was identified as the only significant, independent risk factor in patients aged 60 years or above (OR = 5.7). However, transmission appeared to occur only between patients; there was no evidence for recent transmission between hospital staff and patients.

The frequently cited study by Sepkowitz *et al*. [9] distinguished between medical and non-medical staff, and concluded that the risk of a recent TB transmission for health-care workers was nearly three times as great as for others. However, this study was not based on the general population, as only patients from six hospitals – classified as "hospital staff" or "outside patients" are compared with one another. The professions of almost 30% of the 201 patients were unknown and, among the 20 infected health-care workers, 8 of the 13 cluster members turned out to be HIV-positive. The conclusion of this study, which took place at the height of the New York HIV epidemic, "that many of the apparently sporadic cases of tuberculosis among health-care workers may be due to unrecognised occupational transmission", does not strictly follow from the study's data, and can in any case hardly be applied to European conditions.

Van Deutekom *et al*. [7], in their population-based "Amsterdam" study, were able to assign 47% of 459 patients registered between 1 July 1992 and 1 January 1995 to clusters. They reached a conclusion opposite to that of Sepkowitz *et al*.: only 6 out of a total of 17 patients (8 Dutch, 9 immigrants) working in the health-care sector were cluster members. "Health-care working" thus emerged as a negative predictor of clustering.

Lemaitre *et al*. [8] looked for nosocomial transmission in a Parisian teaching hospital. A total of 161 RFLP isolates were studied. Interestingly, only 5 (13%) of the 40 cluster patients but 34 (28%) of the 121 non-clustered patients had been admitted to hospital before the diagnosis of TB. None of the 40 members of, in all, 12 clusters showed evidence of transmission within the hospital. (However, the study period was relatively short: 1 March 1993 to 30 April 1995, with only sputum-positive patients being investigated up to 28 February 1994.)

The results of the present study indeed confirm strikingly the (relative) impact of health-care employment on recent transmission. Of course, in view of the small number of cases of active disease we cannot conclude that there is a very high risk in acquiring TB in health-care settings. However, a highly significant association between health-care work and clustering was found (Table 2). Most cases (except the employees in clusters B and D) remained unrecognised until the result of RFLP fingerprinting became available. A conspicuous observation in our study was that none of the infections took place in a chest clinic, but rather in general-internal or ENT wards, (in one case, involving an out-patient); thus, in none of these cases had precautions against airborne infection been taken – e.g., an isolation room with negative pressure relative to the surroundings, or use of disposable splash-proofed masks (N95 or HEPA) by persons entering the room. Furthermore, transmission was always between an infected patient and a health-care worker, and never between patients; thus, there were no nosocomial outbreaks. The risk of TB infection for nursing-home employees is believed to be considerably higher in general – a 1990 CDC study reported that it was as much as three times higher [20] – than the rate found in other adult employees of similar age, race, and sex. For this reason, the new German law on infectious diseases (IfSG paragraph 36, section 4) that came into force on 1 January 2001 requires that new residents provide a medical certificate that they are free of TB. Because the law's protective effect on TB infection had been limited only to the last 12 months of our study, it was a considerable surprise that not one case of recent transmission within this sector was recorded in the present study, in spite of its long duration.

It is well known that investigation of hospital contacts is often difficult to conduct because the movement of patients, and the changing work assignments of personnel, make it difficult to assess the nature and extent of contact between an index patient and staff members with actual contact [21]. As our broad, comparative cluster analysis shows, the principal problem is that at the beginning of a patient contact the possibility of a TB infection is not considered. This means that adequate account is not taken of the infectiousness of the index case and – because the diagnosis comes too late – a previous exposure will be forgotten, or only the people with the most frequent exposure will ultimately be identified. It is clearly insufficient to record retrospectively only the co-patients of an index person who shared the patient's room and the nurses or doctors who were directly assigned to care for him/her on the ward. Even employees with brief contact or – as described above – members of the cleaning staff responsible for decontamination may be exposed to infection spread by droplets. According to the classical finding of Wells and Riley [22] that a single viable TB bacillus, once inhaled, is, sufficient to produce infection, the risk of infection is not necessarily a function of duration of contact. Thus, the risk arising from a prolonged period of proximity need not be greater than that arising from a short phase of heightened contagiousness. In our study the risk of clustering is at least about six-fold higher in patients whose diagnosis of TB disease is confirmed more than six months after the beginning of symptoms. Thus, spreading of TB by infectious persons who remain unidentified for an unexpectedly long time may play an important role in TB transmission.

The most crucial prerequisite for effective contact tracing is the verification that (a) a case of infectious TB is reported to the hospital concerned as promptly as possible, and (b) ideally, it should be a matter of routine to note, *before *the disease is diagnosed, as completely as possible who and in which period has looked after the index patient. As in the management of an outbreak, an organised and continuously supervised procedure is necessary, in order to establish the personal, spatial and temporal framework of contact. Above all, the need to obtain a detailed medical history from each patient has to be stressed, especially if the patient has received previous treatment for tuberculosis or belongs to defined high risk groups.

Another reason why it is important to find every contact person is the converse one: not every RFLP cluster will demonstrate a recent epidemiological link [6,23,24]. Owing to the possibility that a portion of clustered patients are infected with circulating strains that are prevalent in a given community, spreading over a long period, or that were imported from endemic areas of the world developed an active TB by chance within the study period, mixed clusters are not uncommon. In these, cases of recent transmission are associated with cluster members between whom there is no epidemiological connection. Examples of this in the present study are clusters B and G, in which the respective transmission rates *n*-1 (i.e., cluster size minus 1) are not 8 and 4, but rather 1 and 0. However, one should take into account the fact that TB infection may occur among highly infectious source cases and their recipients through casual contacts. Thus, a conventional epidemiological investigation – even if it is conducted as meticulously as possible – is unlikely to be able to pick up all of the infected persons, possibly leading to an overestimate of the total proportion of cluster members between whom no epidemiological linkage is assumed.

The predominance of health-care employment as an independent risk factor in this study might have important implications for future tuberculosis control in inner cities. The effectiveness of traditional contact tracing according to the "stone-in-the-pond" principle [25,26] in social risk groups such as alcoholics, drugs addicts or homeless people is obviously largely dependent on their individual willingness to co-operate [27]. Thus, it is not surprising that in Hamburg only approximately 25% (26/103) of clustered patients with confirmed transmission links have been identified by conventional contact tracing up to now in our study. In health-care settings, however, the success of determining transmission links is – as the examples of our clusters show – largely associated with the quality of each case management as defined in current guidelines [28]. This implies that IS*6110 *RFLP typing can help to define more precisely where the deficiencies are, and whose standards have to be adapted in awareness of possible exposure to transmission.

In conclusion, the results of our study demonstrate that health-care workers – even those outside high-risk-settings – are at particular risk of recent TB transmission. Since most cases remained unrecognised, there is a need for improved strategies for contact tracing that avoid ineffective procedures and allow a better-targeted identification of cases. Continual prospective RFLP fingerprinting is essential to assess the efficiency of such new standards, and should therefore play an integral part in communal TB control.

## Competing interests

The author(s) declare that they have no competing interests.

## Authors' contributions

Roland Diel: Conception and design of the study, acquisition, analysis and interpretation of data, drafting and revising of the article, giving final approval to the version to be published

Andreas Seidler: Statistical analysis and interpretation of data, drafting and revising of the article, giving final approval to the version to be published

Albert Nienhaus: Statistical analysis and interpretation of data, drafting and revising of the article, giving final approval to the version to be published

Sabine Rüsch-Gerdes: Interpretation of data, drafting and revising of the article, giving final approval to the version to be published

Stefan Niemann: Conception and design of the study, acquisition, analysis and interpretation of data, drafting and revising of the article, giving final approval to the version to be published
